# Pathogenic bacterial species and the microbiome of cat fleas (*Ctenocephalides felis*) inhabiting flea-infested homes

**DOI:** 10.1371/journal.pone.0341824

**Published:** 2026-01-30

**Authors:** Taylor E. Gin, Charlotte O. Moore, Trey Tomlinson, Grace Wilson, Amiah Gray, Cameron Sutherland, Kamilyah Miller, Krista Li, Michael Canfield, Brian Herrin, Erin Lashnits, Benjamin Callahan

**Affiliations:** 1 Department of Population Health and Pathobiology, College of Veterinary Medicine, North Carolina State University, Raleigh, North Carolina, United States of America; 2 Department of Clinical Sciences, College of Veterinary Medicine, North Carolina State University, Raleigh, North Carolina, United States of America; 3 Department of Diagnostic Medicine and Pathobiology, College of Veterinary Medicine, Kansas State University, Manhattan, Kansas, United States of America; 4 Department of Medical Sciences, School of Veterinary Medicine, University of Wisconsin-Madison, Madison, Wisconsin, United States of America; 5 Animal Dermatology South, New Port Richey, Florida, United States of America; Institut Pasteur de Madagascar, MADAGASCAR

## Abstract

**Background:**

*Ctenocephalides felis* is a common ectoparasite of dogs and cats and can transmit a variety of pathogens including *Bartonella* and *Rickettsia* species. These bacteria, along with the known endosymbiont *Wolbachia*, are well-documented members of the *C. felis* microbiome, but species-level information is limited. Additionally, little is known about the variation in the *C. felis* microbiome in fleas from different sources and when different sequencing methods are applied to the same samples.

**Objective:**

This study aimed to characterize the flea microbiome using both short-read (V3/V4) and long-read (full-length) 16S rRNA gene sequencing, determine whether long-read sequencing improves species-level identification especially in known pathogenic genera, and evaluate differences in microbial composition between fleas collected from cats, dogs, and environmental traps.

**Methods:**

Fleas were collected from cats, dogs, and traps in flea-infested homes in Florida, pooled by source, and sequenced using short- (V3/V4) and long-read (full-length) 16S rRNA gene sequencing. Microbial prevalence and abundance were compared across sequencing approaches. Community composition was evaluated for differences between sources and houses. Candidate members of the flea microbiome were identified based on a combination of prevalence, abundance, and statistical signatures of potential contaminant origin. For *Rickettsia* and *Bartonella*, species-level taxonomic assignments were refined using a phylogenetic approach.

**Results:**

*Wolbachia*, *Rickettsia*, and *Bartonella* were the most prevalent and abundant taxa. *Spiroplasma* was identified as a fourth core member of the flea microbiome. Long-read sequencing enabled better, but not perfect, species-level classification of *Bartonella* and *Rickettsia* compared to short-read sequencing. Important relationships between specific ASVs and flea sources were identified, for example fleas from cats harbored higher abundances of *B. clarridgeiae* and *B. henselae* than fleas from traps.

## Introduction

The cat flea (*Ctenocephalides felis*) is the most common ectoparasite of dogs and cats [1,2], and is known to vector multiple human pathogens, including *Bartonella* and *Rickettsia* species*.* Cat flea infestations of pet dogs and cats are common, especially in warmer climates such as the Southeastern United States, and populations that do not practice flea prevention [3]. A 2016 study of ectoparasites on free-roaming domestic cats in the central United States found 46.5% (313/673) of cats to be infested by *C. felis* [1]. Between 0.4% and 10.2% of dogs and cats presenting to one national (United States) veterinary hospital were found to be infested by fleas at any one time [4]. Other studies report ranges of *C. felis* infection from 35–92.5% of the studied populations [5–7].

The impact of cat flea infestation on dogs and cats ranges from mild to severe. Skin irritation is a nearly universal symptom that can become more serious when the animal has an allergy or when it perpetuates the itch-scratch cycle to the point of self-harm [8,9]. Cat fleas transmit multiple pathogens to cats and dogs, including *Dipylidium caninum*, a tapeworm that can be ingested along with fleas by a grooming animal, *Bartonella* spp., bacteria that often results in asymptomatic carriage but can also lead to systemic disease (bartonellosis), and *Rickettsia* spp., bacteria which can also result in severe febrile illness [10,11].

In humans, cat flea infestations are an obvious nuisance and skin irritant possibly leading to atopic dermatitis, but cat fleas also serve as the vector for zoonotic illnesses. Cat-scratch disease is caused by *Bartonella* spp. that is passed to the human by a cat scratch or other inoculation of infected flea feces through the skin barrier [11]. Cat-scratch disease most often results in painful regional lymphadenopathy, but more severe manifestations such as seizures or prolonged fever of unknown origin have also been reported [12]. Although not the primary vector, *C. felis* has the potential to carry *Rickettsia typhi,* which is passed to humans through flea bites and can result in murine typhus [13–15]. *Rickettsia felis* is also commonly reported in *C. felis* microbiome studies and is known to result in febrile illness in humans [16,17]. Dogs may act as asymptomatic reservoirs of *R. felis* and can serve as a source of infection for fleas, which can then go on to infect other animals or people in shared environments; this highlights the potential threat of infectious disease transmission from cat fleas to humans even in the absence of a cat [18–20]. Given their physiology, biting lifestyle, and frequent proximity to humans, cat fleas have the potential to be an important vector for many other pathogens as well.

The collection of microorganisms living on and in a flea (loosely its “microbiome”) is still not well understood. This is in part because of the low microbial biomass of flea-associated microbes, which challenges common measurement methods. Previous studies agree on certain components of the flea microbiome, with the three main taxa repeatedly observed being *Bartonella*, *Rickettsia* and the endosymbiont *Wolbachia*, but many other microbial taxa have also been reported in various studies [21–26]. We currently have little knowledge about systematic variation in the flea microbiome based on factors such as host animal or environment. Cats and dogs are common pets, and fleas are a common nuisance for pet owners and contain an entire ecosystem of potential pathogens [27,28].

In this study, we sampled fleas from cats, dogs and environmental traps in households with active *C. felis* infestations. We measured the flea microbiome using both short-read 16S (V3/V4) rRNA gene sequencing and full-length 16S rRNA gene sequencing. We characterized the differences in the flea microbiome based on host animal and investigated the potential for full-length 16S sequencing to better speciate the important pathogenic genera of *Rickettsia* and *Bartonella*. We performed a quantitative comparison of microbiome profiles from short- and long-read sequencing approaches and described the typical flea microbiome in our sample population.

## Materials and methods

### Ethical approval

All animal handling and treatment procedures were reviewed and approved by Kansas State University IACUC #4704 and Kansas State University IRB#11060.

### Enrollment

**Fig 1:**
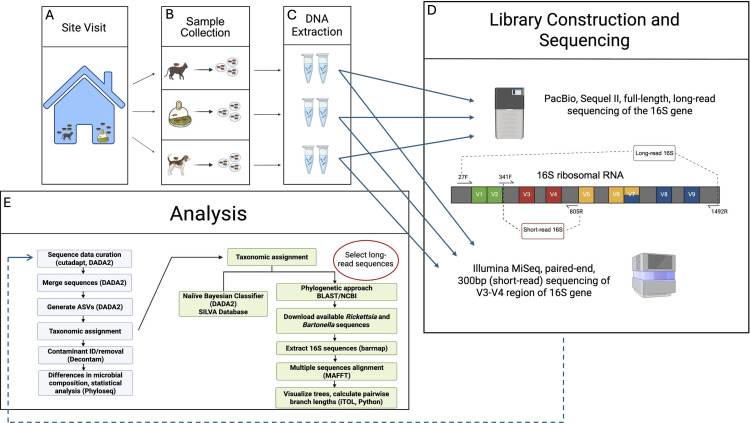
Overview of the workflow from gathering fleas (panels A and B) to DNA extraction (panel C), sequencing (panel D), and analysis (panel E) in a study evaluating short- and long-read 16S rRNA gene sequencing of pooled fleas from different sources (cats, dogs and traps) in flea-infested households in Florida.

This study (“the sequencing study”) was a branch of a primary study (“the efficacy study”) evaluating the efficacy of an orally-administered, adulticidal flea product (lotilaner, Credelio^®^CAT, Elanco Animal Health) in indoor-cat-owning, flea-infested homes in West Central Florida. Homes were evaluated for eligibility in the efficacy study if owners reported a flea infestation in the home, as well as ownership of at least one indoor cat. Up to 10 cats and dogs per home were allowed. Fleas on dogs were also evaluated, and dogs received lotilaner (Credelio^®^). Additional enrollment criteria for the efficacy portion of the study are included in S1 Protocol. Animals did not have to complete the efficacy portion of the study to be eligible for the sequencing portion. Fig 1 provides a diagram of the workflow from flea collection through sequencing.

### Flea collection

At enrollment, homes and animals were evaluated by a team consisting of veterinarians, board-certified veterinary specialists (including one veterinary parasitologist, two small animal internal medicine specialists, and one veterinary dermatologist), and supervised veterinary students and veterinary technicians. At the evaluation for enrollment, flea burden on-host was counted and fleas were collected directly from cats and dogs using a standardized flea combing technique described previously [29,30]. The numbers of adult fleas emerging in each home were also estimated using intermittent light traps as previously described [30,31]. Fleas were taken from three different sources in the home (cats, dogs, traps). To ensure sufficient flea burdens for the efficacy study, on-host fleas were only collected if the animal was host to more than 9 fleas. All fleas were morphologically identified as *Ctenocephalides felis* by a board-certified veterinary parasitologist. Fleas were placed into resealable freezer bags, labeled by the household ID and source (cat, dog, trap), and stored in a -20^o^C freezer.

### Flea pooling and DNA extraction

Fleas were driven on ice from West Central Florida, to the Parasitology Lab at Kansas State University (Manhattan, KS) and divided into pools of three based on household and source. Pools of 3 were then rinsed to reduce the number of environmental surface contaminants that might be present on the outside of the flea that are not representative of the flea microbiome [32]. Briefly, pools of fleas were placed in 500 microliters of 95% EtOH and vortexed for 30 seconds. The EtOH was then removed and the step was repeated using nuclease-free water. The fleas were then removed and placed into ZR BashingBead™ Lysis Tubes (0.1 & 2.0 mm Beads) (Zymo Research; Irvine, CA). The pools were homogenized for 1 minute using a FastPrep-24™ Classic bead beating grinder and lysis system (MP Biomedicals; Santa Ana, CA) and DNA extracted using the QIAwave DNA Blood & Tissue Kit (Qiagen; Hilden, Germany). Twenty (20) controls (labeled EX1-EX20), which consisted of empty Eppendorf tubes, underwent the same DNA extraction process as the flea pools, resulting in a “reagent-only” negative extraction control. Samples were stored at −20ºC until shipment to University of Wisconsin-Madison Biotechnology Center’s DNA Sequencing Facility.

### Library construction and metagenomic sequencing

All sequencing was performed by the University of Wisconsin-Madison Biotechnology Center. Standardized control samples were used according to center’s protocol guidelines, including a positive (a combination of bacterial DNA) and negative (sterile water only) PCR control during the library amplification and indexing PCR steps. Additionally, the center uses 25 cycles for the library amplification PCR and 8 cycles for the indexing PCR. The final concentration of DNA library loaded onto the flow cells was 9pM.

#### Illumina 16S (V3-V4) short-read sequencing.

Purified genomic DNA was submitted to the University of Wisconsin-Madison Biotechnology Center. DNA concentration was verified fluorometrically using either the Qubit® dsDNA HS Assay Kit or Quant-iT™ PicoGreen® dsDNA Assay Kit (ThermoFisher Scientific, Waltham, MA, USA). Samples were prepared in a similar process to the one described in Illumina’s 16S Metagenomic Sequencing Library Preparation Protocol, Part # 15044223 Rev. B (Illumina Inc., San Diego, California, USA) with the following modifications: The 16S rRNA gene V3/V4 variable region was amplified with fusion primers (forward primer 341f: 5’-ACACTCTTTCCCTACACGACGCTCTTCCGATCT(N)_0/6_CCTACGGGNGGCWGCAG-3’, reverse primer 805r: 5’-GTGACTGGAGTTCAGACGTGTGCTCTTCCGATCT(N)_0/6_GACTACHVGGGTATCTAATCC-3’). Region specific primers were previously described in Klindworth et al.[33], 2013 (underlined sequences above), and were modified to add 0 or 6 random nucleotides ((N)_0/6_) and Illumina adapter overhang nucleotide sequences 5′ of the gene‐specific sequences. Following initial amplification, reactions were cleaned using a 0.7x volume of AxyPrep Mag PCR clean-up beads (Axygen Biosciences, Union City, CA). In a subsequent PCR, Illumina dual indexes and sequencing adapters were added using the following primers (forward primer: 5’-ATGATACGGCGACCACCGAGATCTACAC[5555555555]ACACTCTTTCCCTACACGACGCTCTTCCGATCT-3’, Reverse Primer: 5’-CAAGCAGAAGACGGCATACGAGAT[7777777777]GTGACTGGAGTTCAGACGTGTGCTCTTCCGATCT −3’, where bracketed sequences are 10 bp custom Unique Dual Indexes). Following PCR, reactions were cleaned using a 0.7x volume of AxyPrep Mag PCR clean-up beads (Axygen Biosciences). Quality and quantity of the finished libraries were assessed using an Agilent 4200 TapeStation DNA 1000 kit (Agilent Technologies, Santa Clara, CA) and Qubit® dsDNA HS Assay Kit (ThermoFisher Scientific), respectively. Libraries were pooled in an equimolar fashion and appropriately diluted prior to sequencing. Paired end, 300 bp sequencing was performed using the Illumina MiSeq Sequencer and a MiSeq 600 bp (v3) sequencing cartridge.

#### PacBio full-length 16S long-read sequencing.

Purified genomic DNA was submitted to the University of Wisconsin-Madison Biotechnology Center. DNA concentration was verified fluorometrically using either the Qubit® dsDNA HS Assay Kit or Quant-iT™ PicoGreen® dsDNA Assay Kit (ThermoFisher Scientific, Waltham, MA, USA). A Pacific Biosciences HiFi library was prepared according to PN 102-359-000 Version 03 (Pacific Biosciences). Modifications include performing upfront PCR with PacBio 16s primers: **27F (Forward Primer):** 5’-GCATCTAGRGTTYGATYMTGGCTCAG-3’, **1492R (Reverse Primer):** 5’-GCATCRGYTACCTTGTTACGACTT-3’ and KAPA HiFi HotStart ReadyMix (Roche) and a purification using AxyPrep Mag PCR beads prior to pooling. Library quality was assessed using the Agilent TapeStation System. Library was quantified using the Qubit™ dsDNA High Sensitivity kit. The library was sequenced on a Sequel II using Sequel Polymerase Binding Kit 2.2 at the University of Wisconsin-Madison Biotechnology Center DNA Sequencing Facility.

### Analysis

#### Sequence data curation.

Primers were removed from the short-read 16S sequencing data using cutadapt, while for the long-read 16S data the DADA2 R package function `removePrimers` performed this task [34,35]. DADA2 was used to perform quality filtering, error correction, chimera removal and generation of final amplicon sequence variant (ASV) tables. Short-read data processing followed the standard DADA2 tutorial workflow (https://benjjneb.github.io/dada2/tutorial.html), while long-read data processing followed the PacBio HiFi specific workflows associated with the paper validating DADA2 for use with long-read amplicon sequencing data [35]. Sample metadata, taxonomic assignments and ASV tables were merged into a single object using the phyloseq R package [36]. The decontam-frequency method, which makes use of the DNA concentrations of each sample that were measured by Qubit prior to sequencing as described above, was used to score each ASV according to its resemblance to expected patterns of true sequences originating from the flea samples versus contaminants introduced from outside the samples (i.e., a “contaminant origin”) [37]. Taxa that met the following criteria were considered as candidate members of the *C. felis* microbiome: decontam score ≥ 0.5, relative abundance ≥ 0.001, and prevalence ≥ 0.10. Once the candidate taxa were identified, plausibility as a legitimate part of the *C. felis* microbiome rather than a contaminant was further considered through literature evaluation and expert opinion [38–40]. Reproducible scripts for sequence curation are available at: https://github.com/t-gin/fleas_16S.

#### Taxonomic assignment.

For both short-read and long-read 16S data, initial taxonomic assignment was performed by the naïve Bayesian classifier method, using the `assignTaxonomy` implementation available in the DADA2 R package [41]. The SILVA v138.2 database was used as the taxonomic reference [42]. Taxonomic assignments were made when bootstrap confidence was greater than 50%. We followed DADA2 developer guidelines regarding taxonomic assignment which recommend using `assignTaxonomy` with “silva_nr99_v138.2_toGenus_trainset.fa.gz” as the reference database for taxonomic assignment to genus level for short-read 16S data, followed by species assignment using `addSpecies` with the “silva_v138.2_assignSpecies.fa.gz” dataset. For long-read 16S data, we used `assignTaxonomy` with “silva_nr99_v138.2_toSpecies_trainset.fa.gz” as the reference database for taxonomic assignment to species level.

For some full-length 16S sequences, manual species-level assignments were made for ASVs considered as candidates for the *C. felis* microbiome by BLAST-ing sequences against the nt database. For the focal pathogenic genera of *Rickettsia* and *Bartonella,* phylogenetic tree construction was performed to verify BLAST results and find the nearest species.

#### Phylogenetic tree construction.

We downloaded all available *Rickettsia* (501 as of Nov 5, 2024) and *Bartonella* (26 as of Feb 13, 2025) genome assemblies from NCBI (S2 and S3 Tables, respectively). We extracted full-length 16S rRNA gene sequences from the downloaded assemblies using Barrnap [43], and kept only those sequences that fell in the expected length range of 1450–1500 nucleotides. Multiple sequence alignment was performed on the combined set of reference and study full-length 16S sequences using MAFFT [44]. Maximum-likelihood phylogenetic trees were constructed with RAxML and a GTR + G model. The final *Rickettsia* tree was rooted to *Wolbachia pipientis* (S1 Fig), a closely related genus, and the final *Bartonella* tree was rooted to *Rhizobium beringeri* (S2 Fig). The trees were manipulated and visualized using the Interactive Tree of Life (iTOL) platform [45,46]. To determine the closest relatives of each ASV, we computed pairwise branch lengths and the closest reference sequence defined to the species level between all nodes.

#### Differences in microbial community composition between sources.

To evaluate differences in microbial community composition among sample sources (dogs, cats, and traps), beta diversity was calculated using the Bray-Curtis dissimilarity metric. Distance matrices were generated from the ASV abundance table using the `distance` function in the phyloseq package. Ordination was performed using Principal Coordinates Analysis (PCoA) to visualize beta diversity. Statistical differences in community composition were assessed using PERMANOVA (Permutational Multivariate Analysis of Variance) via the `adonis2` function in the vegan R package [47]. To account for unequal numbers of flea pools per household, permutations were constrained within households using the `strata` argument. The effects of household and sample source were tested in a sequential manner: Dissimilarity ~ Household + Source

Significance was determined using 9,999 permutations, and the proportion of variance explained by each factor was quantified (R²). Pairwise PERMANOVA comparisons among sources were conducted to identify specific group differences.

#### Statistical analysis.

**Fig 2:**
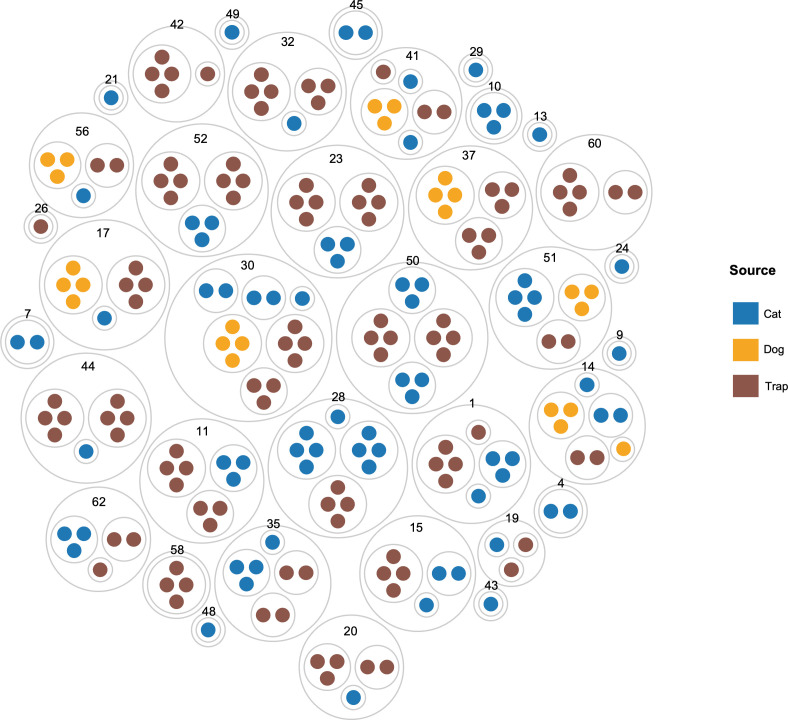
Circle packing diagram of the flea pools sequenced by household and source. Outer gray circles represent a household and are labeled by house number. Inner gray circles represent a source (cat, dog, or trap) within the household, and the inner colored circles represent the number of pools sequenced from that source. Ex: Household 15 includes 1 sampled trap with 4 sequenced pools, and 2 sampled cats, one cat with 1 sequenced pool and a second cat with 2 sequenced pools.

We performed two-sided Wilcoxon rank sum tests to find differences in the relative abundances of specific ASVs and taxa between sources. We tested dog versus cat, dog versus trap, cat versus trap, and fleas from households with dogs to fleas from households with no dogs. Tests were performed at both the ASV and genus level. Unless otherwise stated, all figures were generated in R. Fig 1 was made using BioRender [48]. A publication license is available upon request. Fig 2 was created using RAWGraphs [49].

## Results

### Study design and sample data

We collected fleas for sequencing from 36 homes in the Tampa, Florida, area that contained at least one indoor-only cat with fleas and that voluntarily enrolled in a wider study testing the efficacy of lotilaner for flea infestations (see Methods). There was wide variability in other household characteristics, including the number and type of other animals in the household and the intensity of the flea infestation. Fleas were collected from three sources: Directly from the cat(s) in the household (n = 40 cats), the dog(s) in the household (n = 8), and from overnight traps (n = 40). Details on the per-household sampling are reported in Fig 2 and S4 Table. Fleas were sampled by combing cats and (when present) dogs residing in these households [29]. Flea traps were placed in households overnight and captured fleas were sampled the next day, as previously described [31]. Due to low microbial biomass, all DNA extractions were performed on pools of 3 fleas collected from the same source (cat, dog or trap) within a household. Two different methods were then used to amplify and sequence the 16S rRNA gene (Methods): Short-read Illumina sequencing of the V3-V4 region, and long-read PacBio HiFi sequencing of the full-length 16S rRNA gene. In total, we sequenced 74 pools collected from cats, 25 pools collected from dogs and 113 pools collected from traps. An overview of the read counts across samples at each stage of our Methods is available in S1 Table.

### Species-level classifications are made from long-read 16S rRNA gene sequencing

**Fig 3:**
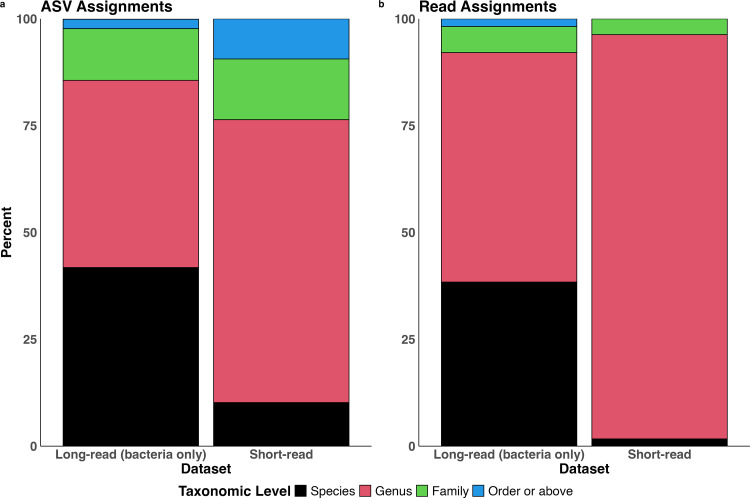
Proportion of (a) bacterial ASVs and (b) bacterial reads classified to different terminal taxonomic levels from short-read and long-read 16S rRNA gene sequences. Short- and long-read 16S sequencing data was generated from the same set of pooled flea samples. Automated taxonomic assignment using the Silva reference database was performed in the DADA2 R package following the developer recommendations.

More species were identified in the long-read than short-read data using the Silva database and taxonomic assignment with DADA2 R package (Methods, Fig 3). There were 9,708,783 reads and 1,401 ASVs generated from short-read sequencing, all of which were assigned to the kingdom Bacteria. There were 4,310,036 reads and 2,616 ASVs generated from the long-read sequencing, but a substantial fraction (~60%) of the ASVs were assigned to the Kingdom Eukaryota and were determined to be off-target amplification of the host *C. felis* genome (18S rRNA gene) by the 27F/1492R primer set. These Eukaryotic reads/ASVs were removed prior to all subsequent analysis, yielding 3,445,891 bacterial reads and 1,047 bacterial ASVs generated from long-read sequencing.

For short-read ASVs: 10.2% were identified to the Species level, 66.2% to the Genus level, 14.2% to the Family level, and 131 (9.4%) to Order, Class, Phylum, or Kingdom. For long-read ASVs: 41.8% were assigned to the Species level, 43.8% to the Genus level, 12.1% to the Family level, and 2.2% to Order or higher. The higher proportion of species-level assignments was even more apparent when weighting ASVs by their abundance (equivalent to considering assignment at the read-level): 38.4% of long reads were assigned to the species level versus only 1.7% of short reads.

### *Wolbachia*, *Rickettsia*, *Bartonella,* and *Spiroplasma* comprise the majority of the *C. felis* microbiome

**Fig 4:**
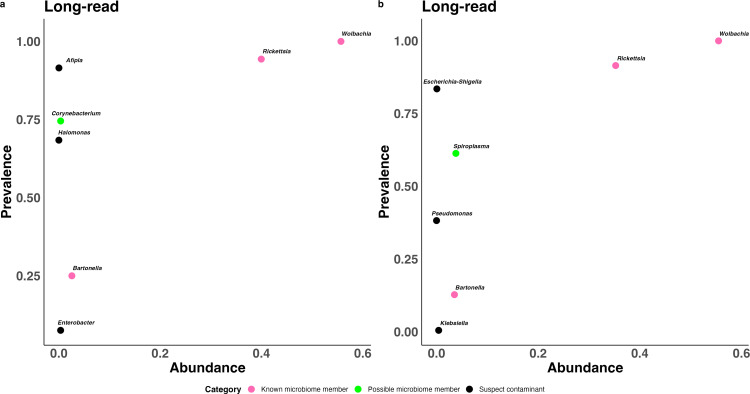
The prevalence and abundance of the top 5 most abundant and prevalent genera following short- and long-read 16S rRNA gene sequencing. Points are colored based on the categorization of genera as known microbiome members (pink), possible microbiome members (green), or suspect contaminants (black). Known microbiome members include the well-documented taxa *Wolbachia*, *Rickettsia*, and *Bartonella*. Possible microbiome members and candidate taxa were designated as such following evaluation of decontam scores and scrutiny of literature describing taxa identified in prior *C. felis* sequencing studies, as well as literature describing common contaminants in microbiome studies.

The most prevalent and most abundant genera across both sequencing types (Fig 4) prominently included the three previously agreed upon members of the *C. felis* microbiome (plotted in pink): *Rickettsia*, *Wolbachia*, and *Bartonella*. In both the short-read and long-read data, *Wolbachia* and *Rickettsia* were the two most prevalent genera and, by far, the two most abundant genera (together 91.1% of short reads, 88.3% of long reads). *Bartonella* prevalence was more modest (1.9% in short-read data, 4.3% in long-read data), but its abundance was third-highest in the short-read and fourth-highest in the long-read data.

One genus outside the established three had a notably high abundance: *Spiroplasma* (Fig 4, plotted in green). *Spiroplasma* represented the third-most-abundant genus at 3.0% of all long reads; a higher abundance than *Bartonella*. *Spiroplasma* was the only genus outside the established three to exceed a study-wide abundance of 0.43% in either dataset. Although *Spiroplasma* was nearly absent in the short-read data with a study-wide abundance of only 0.018% (1,795/9,708,783 reads), this is likely because of poor amplification efficiency of *Spiroplasma* by the V3/V4 primer set. The 341F primer used to amplify the V3/V4 sequences here had at least two mismatches to every full-length *Spiroplasma* ASV recovered by the long-read sequencing.

### Contaminant evaluation supports *Spiroplasma* as another member of the *C. felis* microbiome

We expanded our evaluation of microbial taxa as potential members of the *C. felis* microbiome by scoring ASVs for potential contaminant origin and by consulting the prior literature. The decontam-frequency method uses the patterns of ASV abundance versus input DNA concentration to produce a score between zero and one, with low scores near zero indicating consistency with contaminant origin and high scores near one indicating consistency with the ASV being truly present in the samples [37]. We also reviewed the literature including *C. felis* microbiome studies, manuscripts evaluating and describing common contaminants in microbiome research generally, and studies more narrowly focused on specific taxa of interest.

**Fig 5:**
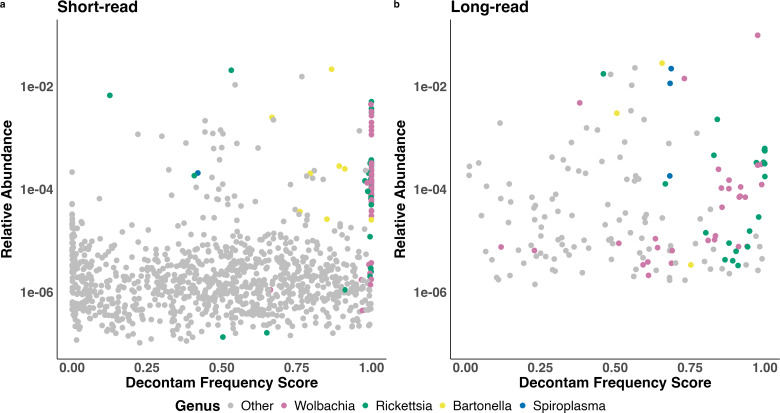
Relative abundance and decontam frequency score for all bacterial ASVs present in two-or-more samples following short- (left) and long-read (right) 16S rRNA gene sequencing. Decontam scores range from 0 to 1 with high scores suggesting true sample origin and low scores suggesting contaminant origin. Colors indicate known core taxa (*Wolbachia* in pink, *Rickettsia* in green, *Bartonella* in yellow) and a proposed fourth core taxa (*Spiroplasma* in blue) based on its high prevalence, abundance, and decontam frequency score in the long-read dataset.

ASVs with high decontam scores (consistent with true presence in the sample) were highly enriched for *Wolbachia*, *Rickettsia* and *Bartonella* (Fig 5). In both short- and long-read data, and especially in the short-read data, the highest scoring ASVs were dominated by ASVs from the established three members of the *C. felis* microbiome, suggesting that decontam scoring worked effectively in our data. Median decontam scores (Table 1) for ASVs from *Wolbachia*, *Rickettsia* and *Bartonella* all were clearly higher than 0.5, suggesting better concordance with true sample origin than contaminant origin.

**Table 1:** Decontam scores, prevalences (presence or absence in a tested pool), and abundances for core and candidate taxa found in short- and long-read 16S sequencing. Scores were defined by the decontam-frequency method at the ASV level. Scores range from 0 to 1 with high scores suggesting true sample origin and low scores suggesting contaminant origin. For genera, the range and median are across all ASVs assigned to that genus.

	Long-read				Short-read			
**Genus/ASV**	**Decontam Range**	**Decontam Median**	**Abundance**	**Prevalence**	**Decontam Range**	**Decontam Median**	**Abundance**	**Prevalence**
***Wolbachia***	0.12 - 0.99	0.80	55%	100%	0.66 - 1	1.0	55%	100%
***Rickettsia***	0.46 - 1	0.93	35%	92%	0.13 - 1	1.0	40%	94%
***Bartonella***	0.51 - 0.75	0.66	3.5%	13%	0.67 - 1.0	0.86	2.6%	25%
***Spiroplasma***	0.68 - 0.68	0.68	3.8%	61%	0.42	0.42	0.023%	55%
***Afipia***	N/A	N/A	0%	0%	0 - 0.04	0.02	0.023%	92%
***Corynebacterium***	0.23-0.60	0.53	0.43%	26%	0 - 0.94	0.33	0.36%	75%
***Halomonas***	N/A	N/A	0%	0%	0 - 0.60	0.015	0.00079%	68%
***Enterobacter***	N/A	N/A	0.0052%	1.4%	0.44 - 0.49	0.46	0.36%	7.5%
***Escherichia- shigella***	0.014-0.53	0.10	0.077%	83%	0 - 0.66	0.33	0.0026%	35%
***Pseudomonas***	0.30-0.60	0.45	0.013%	38%	0 - 0.96	0.11	0.0066%	62%
***Klebsiella***	N/A	N/A	0.43%	0.47%	0.65	0.65	0.0046%	2.8%
**Long-read ASV 7 (Family Neisseriaceae*)***	0.57	0.57	2.3%	24%	N/A	N/A	N/A	N/A
**Long-read ASV 21 *(Corynebacterium falsenii)***	0.60	0.60	0.23%	15%	N/A	N/A	N/A	N/A

Decontam scores and prior literature support *Spiroplasma* as another member of the cat flea microbiome. *Spiroplasma* was essentially undetected in the short-read data probably due to poor amplification by the V3-V4 primer set, so here we only consider its long-read data. The three *Spiroplasma* ASVs present in more than one sample all had decontam scores greater than 0.5, indicating an abundance pattern more consistent with true presence in the sample than contamination, and the median decontam score of 0.68 was effectively equal to the median decontam score of 0.66 for established flea microbiome member *Bartonella*. These statistical patterns are further buttressed by previous reports of *Spiroplasma* in the *C. felis* microbiome [22,24,50], the established presence and functionality of *Spiroplasma* in other insects [51,52], and the high abundance of *Spiroplasma* in the long-read data.

In contrast, decontam scores and prior literature mostly argued against other high prevalence genera as true members of the cat flea microbiome (Fig 4; Table 1). *Afipia* and *Halomonas* had very high prevalence in the short-read data (92% and 68%) but low abundances (0.023% and 0.0079%), were not identified in the long-read data, and had extremely low decontam scores (median = 0.02; median = 0.015) strongly indicating consistency with contaminant origin. *Escherichia-Shigella* and *Pseudomonas* had high prevalence in the long-read data (83% and 38%) but low abundances (0.077% and 0.013%), median decontam scores less than 0.5 in both short- and long-read datasets (Table 1), and are commonly reported contaminants in microbiome studies [38]. *Enterobacter* was the fifth most abundant genus (0.36%) in the short-read data, but its prevalence was only 7.5% and its median decontam score of 0.46 was below the 0.5 threshold. It had low prevalence (1.4%) and low abundance (0.0052%) in the long-read data. *Klebsiella* was the fifth most abundant genus (0.43%) in the long-read data, but it had a very low prevalence of 0.47% (1/212 samples) and was not assigned a decontam score, as decontam-frequency scores are not assigned when a taxa appears in only one sample [37]. In the short-read data, *Klebsiella* was neither highly prevalent (2.8%) nor abundant (0.0046%) and did not get assigned a decontam score. Species of *Klebsiella* have previously been reported in at least two *C. felis* studies but neither study was able to convincingly prove it as a microbiome candidate [22,53]. To the author’s knowledge, *Enterobacter* has not been described in *C. felis* studies, and neither *Enterobacter* nor *Klebsiella* are reported as common microbial contaminants. *Corynebacterium* had a prevalence of 75% in the short-read data and 26% in the long-read data, and had a median decontam score of 0.53 in the short-read data, slightly above the 0.5 mark. However, its median decontam score in the long-read data was just 0.33, and its study-wide abundances were only 0.36% in the short-read data and 0.43% in the long-read data. *Corynebacterium* commonly inhabits the animal skin habitat, making it a plausible contaminant contributed at the time of sampling, but it has also been described in *C. felis* sequencing studies before and after washing the fleas, which could indicate it is ingested by fleas inhabiting cats and dogs [54,55].

We expanded our genus-level evaluation of potential members of the cat flea microbiomes to all long-read ASVs with decontam scores ≥0.5, relative abundances ≥0.1% and prevalences ≥10%. Only two ASVs met these thresholds: long-read ASV 7 identified as Family Neisseriaceae and with a best-hit match with 93.6% identity to *Snodgrassella alvi* via BLAST, and long-read ASV 21 identified as *Corynebacterium falsenii* (97.51% identity with BLAST). *Snodgrassella* is a known member of honeybee microbiomes, and has also been identified in other *C. felis* sequencing studies [24,26,56]. *Corynebacterium* has also been detected in a small number of *C. felis* sequencing studies, but it has also been implicated as a common contaminant in low-biomass microbiome studies such as ours [38,53,57]. Both of these taxa warrant further investigation and are considered more closely in the Discussion. The sequences representing these two taxa in our dataset are available in S1 File.

### Long-read sequencing indicates the presence of pathogenic species of *Bartonella* and *Rickettsia* inhabiting cat fleas

*Rickettsia* and *Bartonella* are genera that contain multiple pathogenic species that can infect animals and/or humans, but the nature of this threat depends on the specific bacterial species. Given the increased potential for species-level identification from full-length 16S sequencing, we further investigated the species-level identities of *Rickettsia* and *Bartonella* ASVs in the long-read data. Two methods were used to identify the species of these important taxa, the results of which are summarized in Table 2. There were 61 long-read ASVs identified as *Rickettsia*, of which 14 were identified to the species level using DADA2’s implementation of the naïve Bayesian classifier method with the SILVA database v138.2 [35,42]. Eight (8) *Rickettsia* ASVs were identified as *Rickettsia asembonensis* and 6 were identified as *Rickettsia japonica*. *R. japonica* has not previously been identified in North America. We performed a BLAST search for all *R. japonica* ASVs against the BLAST nt database and found unclear results consisting of close matches to multiple Rickettsial species. Therefore, to better classify all of the Rickettsial ASVs, we constructed a phylogenetic tree from the 16S rRNA gene sequences from *Rickettsia* reference genomes (Methods) and then identified the closest phylogenetic relatives to the Rickettsial ASVs in our dataset (Methods). Using this approach, 43/61 *Rickettsia* ASVs were identified as *Rickettsia felis*, a known pathogenic species, and the remaining 18/61 were identified as *Rickettsia tillamookensis*. These results and phylogenetic distances are available in S5 Table. All 6 of the Rickettsial ASVs initially assigned to *R. japonica* were reclassified as *R. felis* by this more detailed and customized approach, and of the 8 ASVs assigned to *R. asembonensis*, 6 were reassigned to *R. felis*, and 2 were reassigned to *R. tillamookensis*.

**Table 2:** Summary of species-level taxonomic assignments between two different methods for *Rickettsia* and *Bartonella* ASVs generated from long-read/full-length 16S sequencing of the *C. felis* microbiome.

Taxa	Read assignments (ASVs), naïve Bayesian classifier	Read assignments (ASVs), phylogenetic approach
***R. japonica***	1,011,218 (6)	0 (0)
***R. asembonensis***	36 (8)	0 (0)
**No species assignment (*Rickettsia*)**	92,005 (47)	0 (0)
***R. felis***	0 (0)	1,018,593 (43)
***R. tillamookensis***	0 (0)	84,666 (18)
***B. clarridgeiae***	130,600 (10)	130,593 (9)
***B. henselae***	18,710 (3)	14,063 (1)
***B. koehlerae***	3 (0)	4661 (5)
**No species assignment (*Bartonella*)**	4 (2)	0 (0)

Fifteen long-read ASVs were identified as *Bartonella* using the naïve Bayesian classifier method with the SILVA database. Of these, ten were classified as *Bartonella clarridgeiae*, two as *Bartonella henselae*, one as *Bartonella koehlerae*, and two were not classified to the species level. Similar to the Rickettsial ASVs, BLAST results consisted of close matches to multiple species. To refine these assignments, we again applied our customized phylogenetic approach on each *Bartonella* ASV. The closest phylogenetic relatives were identified as *Bartonella clarridgeiae* (9/15 ASVs), *Bartonella koehlerae* (5/15 ASVs), and *Bartonella henselae* (1/15 ASVs). Five ASVs were discordantly classified between the two methods. Notably, one ASV (ASV 30), which comprised a relatively high proportion of all *Bartonella* reads (3.1%), was initially identified as *B. henselae* by the naïve Bayesian classifier and SILVA database but was identified as *B. koehlerae* using our phylogenetic approach. Additionally, two ASVs initially assigned to *B. clarridgeiae* were more closely related phylogenetically to *B. koehlerae*, and of the two ASVs that could not be classified to the species level, one was most closely related to *B. koehlerae* and the other to *B. clarridgeiae*. Phylogenetic distances for *Bartonella* are available in S6 Table.

### Household and host type are statistically significant but modest influences on the flea microbiome

#### Community composition differs significantly but not substantially between cats, dogs and traps.

**Fig 6:**
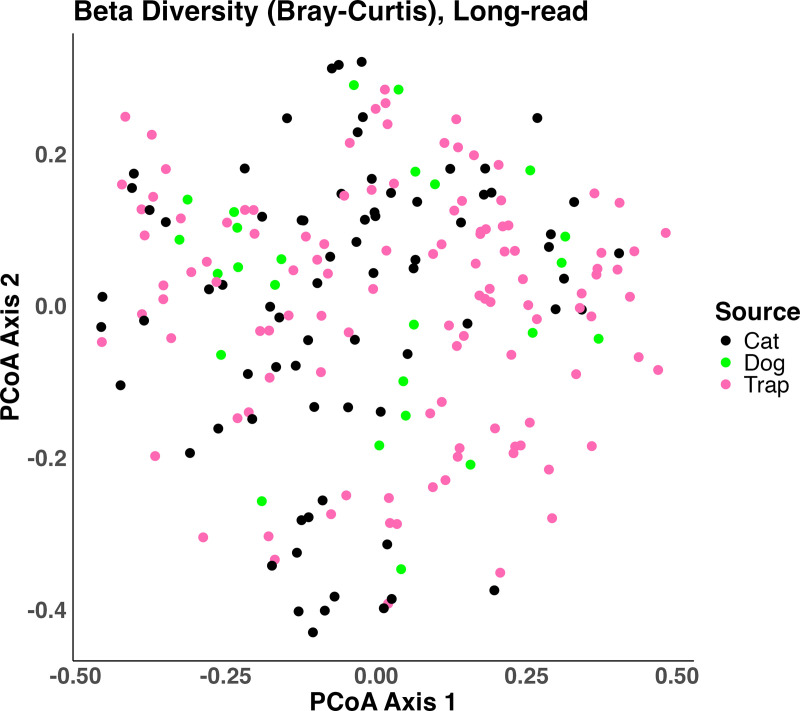
PCoA plot depicting lack of major effect from any one source (dog, cat, or trap) on community composition following long-read 16S rRNA gene sequencing of flea pools from different sources in flea-infested homes in Florida.

We compared the measured microbial communities between flea sources (cats, dogs, and environmental traps) and households using an approach called beta-diversity analysis, which separates the differences between communities from the diversity within communities (Fig 6). Beta diversity analysis revealed statistically significant differences in microbial community composition between flea sources (dog, cat, or trap) that explained 2.67% of the variation in community composition (R² = 0.02668, p-value = 0.0006). Having a dog in the household explained 1.76% of the variation in community composition (R² = 0.0176, p-value = 0.001). Household also significantly influenced microbial community composition, explaining an additional 3.4% of the variation (R² = 0.34, P < 0.0001). However, while each of these factors reached the commonly accepted level of statistical significance (P < 0.05), their effect-size was small (~1–3%) indicating that most of the variability in measured microbiome composition between flea pools is not explained by these factors.

#### Different abundances of ASVs from dogs, cats and traps.

We conducted a two-sided Wilcoxon rank-sum test to assess significant differences in the relative abundance of ASVs across each of the sources, including comparisons between the relative abundance of ASVs found in fleas from dogs vs.cats, dogs vs.traps, cats vs.traps, and sources in households with dogs vs. without dogs (Table 3). ASV 7 (Neisseriaceae) was significantly more abundant in fleas from cats compared to those from dogs (p = 0.000043) or traps (p = 0.000073). Similarly, ASV 6 (*Bartonella clarridgeiae*) was more abundant in fleas from cats compared to those from dogs (p = 0.019) or traps (p = 0.000082). ASV 19 (*Bartonella henselae*) was also significantly more abundant in fleas from cats than in those from traps (p = 0.0011). Fleas from cats exhibited a higher relative abundance of ASV 21 (*Corynebacterium falsenii*) compared to those from dogs (p = 0.0061), while ASV 4 (*Wolbachia* sp.) was significantly more abundant in fleas from cats (p = 0.0042) and dogs (p = 0.0065) than in fleas from traps.

**Table 3:** Top 5 results of two-sided Wilcoxon rank sum tests evaluating the differential abundance of long-read ASVs between sources (cats, dogs, and traps) and between households with and without dogs.

Test	ASV	Taxa	P-value	Direction of outcome
**Dog versus cat**	ASV 7	Neisseriaceae	0.00043	Cat > Dog
	ASV 9	Holosporaceae	0.0054	Dog > Cat
	ASV 21	*Corynebacterium falsenii*	0.0061	Cat > Dog
	ASV 14	*Spiroplasma ixodetis*	0.0071	Dog > Cat
	ASV 6	*Bartonella clarridgeiae*	0.019	Cat > Dog
**Dog versus trap**	ASV 9	Holosporaceae	0.0018	Dog > Trap
	ASV 4	*Wolbachia* sp.	0.0065	Dog > Trap
	ASV 5	*Wolbachia* sp.	0.016	Dog > Trap
	ASV 21	*Corynebacterium falsenii*	0.028	Trap > Dog
	ASV 14	*Spiroplasma ixodetis*	0.033	Trap > Dog
**Cat versus trap**	ASV 6	*Bartonella clarridgeiae*	0.000082	Cat > Trap
	ASV 7	Neisseriaceae	0.00073	Cat > Trap
	ASV 19	*Bartonella henselae*	0.0011	Cat > Trap
	ASV 4	*Wolbachia* sp.	0.0042	Cat > Trap
	ASV 25	Neisseriaceae	0.013	Cat > Trap
**Fleas from households with dogs versus households without dogs**	ASV 9	Holosporaceae	0.000071	Dog > No dog
	ASV 7	Neisseriaceae	0.00028	No dog > Dog
	ASV 21	*Corynebacterium falsenii*	0.00065	No dog > Dog
	ASV 14	*Spiroplasma ixodetis*	0.0019	Dog > No dog
	ASV 1	*Rickettsia felis*	0.0045	Dog > No dog

Fleas collected from households with dogs exhibited higher relative abundances of ASV 9 (Holosporaceae) (p = 0.000071), ASV 14 (*Spiroplasma ixodetis*) (p = 0.0019), and ASV 1 (*Rickettsia felis*) (p = 0.0045) compared to fleas from households without dogs. Conversely, ASV 7 (Neisseriaceae) (p = 0.00028) and ASV 21 (*Corynebacterium falsenii*) (p = 0.00065) were more abundant in fleas from households without dogs.

## Discussion

We used two types of 16S rRNA gene sequencing to analyze the microbes from cat fleas (*C. felis*) collected from flea-infested homes: Short-read (V3/V4) sequencing using Illumina, and long-read (full-length) 16S sequencing using PacBio. We unsurprisingly confirmed *Wolbachia*, *Rickettsia*, and *Bartonella* as dominant members of the *C. felis* microbiome. Through analysis of prevalence, abundance, and decontam scores, we proposed *Spiroplasma* as a fourth core member. We identified two additional candidate taxa – long-read ASVs classified as *Corynebacterium falsenii* and as Family Neisseriaceae – worthy of further investigation as potential members of the cat flea microbiome. We validated the use of short-read (V3/V4) for detection of *Wolbachia*, *Rickettsia*, and *Bartonella* in the *C. felis* microbiome but demonstrated likely primer bias which resulted in lack of *Spiroplasma* detection in the short-read (V3/V4) dataset. Long-read sequencing results and careful review of taxonomic assignments were used to identify pathogenic species of *Bartonella* and *Rickettsia*, underscoring the public health implications of flea infestations in domestic environments. Lastly, we detected differences in microbial composition between fleas from different sources (dogs, cats, or traps) indicating what might be an important ecological relationship between flea source (dog, cat, or environment) and the presence or absence of specific taxa in the *C. felis* microbiome.

*Spiroplasma* has previously been reported in *C. felis* microbiome studies but not described as a core member of the *C. felis* microbiome [22,24,50–52]. In fact, a 2024 study by Moore et. al. hypothesizes that *Spiroplasma* may have previously been misreported as hemotropic *Mycoplasma* in previous flea studies due to its amplification by *Mycoplasma* primers and subsequent misidentification [50]. We did not identify *Mycoplasma* in our long-read data, but did find compelling evidence for *Spiroplasma* as more than just a contaminant or sequencing error. Notably, it was only in our long-read dataset that we clearly identified *Spiroplasma*, likely because of a primer mismatch between *Spiroplasma* and the V3/V4 primer set used in our study and in many others. *Spiroplasma ixodetis*, the putative *Spiroplasma* species identified in our study, has been described as a “facultative endosymbiont” in ticks and not associated with known pathogenesis in any host [52]. At least 45 *Spiroplasma* type-strains have been identified in a variety of plant and arthropod hosts, including ants, moths, beetles, honey bees, wasps, butterflies, and more [51]. Of these 45 *Spiroplasma* type-strains, 7 have been associated with pathogenesis across arthropods (2/7), plants (4/7), and mice (1/7). To the authors’ knowledge, *Spiroplasma* has not been heavily investigated in fleas, and while it’s possible *S. ixodetis* is the *Spiroplasma* species in our study, the 98.41% nucleotide identity between the full-length *Spiroplasma* ASV detected in our study and the *S. ixodetis* 16S rRNA gene reference sequence is not sufficient to conclusively determine that these sequences belong to the same species [58–61]. We may have *S. ixodetis* in our dataset, or we may have another species unique to the flea which has yet to be described. Since *Spiroplasma* is not known to be pathogenic to animals, we chose not to elaborate on species-level information for this genus.

We also identified two low-abundance taxa, Neisseriaceae (ASV 7) and *Corynebacterium falsenii* (ASV 21), as potential members of the *C. felis* microbiome. Neisseriaceae is a large family that comprises many possible taxa, ranging from taxa present in the oral microbiome of dogs and cats (*Neisseria animaloris*), to known insect endosymbionts (*Snodgrassella alvi*), to known human pathogens (*Neisseria gonorrhoeae*). The sequence associated with ASV 7 was found to be most similar to *Snodgrassella alvi*, but only at 93.6%, which is not close enough to definitively report it as this specific organism. Not only did we find a significant signal indicating the presence of a member of Neisseriaceae, but we found that the ASV associated with this family was strongly associated with fleas collected from cats. We hypothesize that this is either the result of increased grooming by cats with fleas (resulting in transfer and uptake of members of the feline oral microbiome), or we could be detecting another *C. felis* endosymbiont that has yet to be described and thrives under the conditions established when the flea has a feline host. We also found evidence for *Corynebacterium falsenii* as a *C. felis* microbiome member. Similar to Neisseriaceae, *Corynebacterium* has been identified in prior flea sequencing studies [53,57]. However, it has also been described as a common contaminant in microbiome sequencing studies, especially those that are considered low-biomass, a term that would aptly describe our study [34]. Interestingly, *C. falsenii* was significantly more abundant in fleas from households without dogs, though the meaning of this finding is unclear. The sequence associated with *C. falsenii* was found to have only 97.5% identity (via nt BLAST) to known *C. falsenii* sequences. *Corynebacterium* has been documented in both cat and dog microbiome studies and may be present under both normal and diseased conditions [54,55,62]. One of the requirements for enrollment was the presence of a cat, so every flea in this study came from a household with a cat but not necessarily a household with a dog.

Here, we were particularly interested in making species-level determinations within the often pathogenic *Bartonella* and *Rickettsia* genera. However, we did not always observe the same result from using a standard taxonomic assignment method adopted from short-read 16S rRNA gene sequencing – the naïve Bayesian classifier and SILVA database implemented in DADA2 – with what we observed when BLAST-ing the full-length sequence by hand. Furthermore, the naïve Bayesian classifier plus SILVA only classified 41.8% of the long-read 16S ASVs to the species level, perhaps due to the conservative bootstrapping approach used to assess confidence by this method and by the presence of closely related named species in key members of the flea microbiome such as *Rickettsia*. By implementing a custom solution based on building genomic phylogenies within *Bartonella* and *Rickettsia*, and then placing the full-length ASVs into those phylogenies, we were able to obtain more robust species-level assignments. Species identification of *Bartonella* is a known challenge, and prior research demonstrates the need for a multipronged approach to determine if one or more species of *Bartonella* are present [63,64]. *Rickettsia* is similar. Overall, our results support the utility of full-length 16S sequencing for studies prioritizing pathogen identification, particularly in cases where closely related species share high sequence similarity within the 16S rRNA gene, but also show that even with full-length 16S sequencing, assignment of closely related pathogenic species might require a more customized approach than the standard tools that were developed for primarily genus-level assignment to short-read 16S rRNA gene sequencing data.

Pre-sequencing factors seemed to play an important role in our study. First, the primer set we used (27F/1492R) to amplify the full 16S rRNA bacterial genome resulted in off-target amplification of the *C. felis* 18S rRNA gene. In the long-read data, 60% of the total ASVs, and more importantly 20% of the total reads, were actually off-target amplification of the *C. felis* 18S rRNA gene. A similar pattern of 18S rRNA gene amplification has been described with this same primer set in coral and demonstrates the importance of primer selection even when “universal primers” are being used [65]. Future studies of the *C. felis* microbiome might benefit from closer inspection of primer sets using local alignment tools or by designing bacteria-specific protocols or primers that lessen off-target amplification [66,67]. For *C. felis* microbiome studies, primer evaluation should also include detection of the primary members of the *C. felis* microbiome: *Wolbachia*, *Rickettsia*, *Bartonella*, and *Spiroplasma*. Blocking primers could also be utilized to allow for the use of common bacterial primers but prevent cross-amplification of non-target DNA [68,69]. Despite a relatively high abundance (3.8%), prevalence (61%), and decontam score (0.62) in the long-read data, *Spiroplasma* was not convincingly detected in the short-read (V3/V4) data, which we suspect is due to primer mismatches rather than a failure of the sequencing platform. An ideal primer set for *C. felis* microbiome sequencing studies would include the ability to detect a broad-range (if not all) bacteria present in the sample, avoid detection of off-target/host DNA, and provide distinct species-level information about the pathogenic taxa, in our case *Rickettsia* and *Bartonella*. For the latter criteria, it is also reasonable to consider post-sequencing protocols that identify species-specific traits for each of these taxa rather than a primer set that has both extreme sensitivity (detects all bacteria present) and specificity (no off-target amplification of host DNA and no identification of species which aren’t actually present in the sample).

We detected higher relative abundances of *B. clarridgeiae* and *B. henselae* in fleas from cats compared to fleas from dogs and traps, respectively. This corresponds to the important ecological role cats play in *Bartonella* transmission, as all three species of *Bartonella* that we identified are associated with disease in humans as well as pet dogs and cats, and are known to have cats as a primary reservoir host [70,71]. *B. clarridgeiae* is known to cause Cat Scratch Disease in humans, including lymphadenopathy, fever, and skin lesions [72]. *B. henselae* has been associated with Cat Scratch Disease, is one of the *Bartonella* species most commonly associated with endocarditis in dogs and humans and can be challenging to diagnose via routine blood culture [73]. It has also been implicated as a cause of bacillary angiomatosis, peliosis hepatis, and other systemic manifestations [74]. *B. koehlerae* has been associated with endocarditis [75]. In general, *Bartonella* can be very challenging to treat due to its intracellular nature and ability to survive despite the presence of antimicrobials [76]. The genus *Rickettsia* similarly encompasses a wide range of pathogenic bacteria, one of which we identified in our study: *R. felis. R. felis* has been described to cause infections in mammals, including people, ranging in signs from high fever to pain (myalgia) and skin lesions [77]. We identified a higher relative abundance of *R. felis* in fleas from households with dogs compared to fleas from households without dogs, which could correspond to recent research suggesting that dogs may be important asymptomatic reservoirs of *R. felis* and able to transmit *R. felis* to *C. felis* without any sign of illness [18,78]. Whether or not dogs are a significant source of *R. felis* transmission, fleas (*C. fellis*) are a known reservoir for *R. felis* and pose a threat regardless of whether a dog is present in the home [77,78]. With all the pathogenic species identified, it is critical for pet owners to understand the hazards of a flea-infested environment when living with cats and dogs, as all of these organisms may pose a threat to the health of humans and animals alike [79].

A major strength of this study was the use of both short- and long-read sequencing, allowing for a direct comparison of taxonomic resolution and detection biases. Our results highlight the need for careful primer selection to capture key members of the flea microbiome (at least including *Wolbachia, Rickettsia, Bartonella, Spiroplasma*) and to avoid amplification of flea host DNA. Our results also show the need for sequencing and bioinformatic approaches tailored to specific research goals, especially if species-level identification within pathogenic genera is desired. We recommend long-read 16S rRNA gene sequencing for studies prioritizing pathogen identification, but with potential modifications to the primer set we used here to reduce off-target amplification of the cat flea genome.

Our study was limited by the use of pooled flea samples, preventing individual-level variation assessments. While we identified significant microbial differences between flea sources, the modest effect sizes suggest that more detailed host and environmental metadata may be needed to fully explain flea microbiome variability. We performed an ethanol rinse of the fleas prior to DNA extraction but did not perform light bleach washing. This may be considered a limitation, as bleach washing of ticks has been shown to reduce external bacterial contamination in microbiome studies [80]. An additional limitation was that we did not include positive controls, which could be incorporated in future studies to evaluate amplification bias and/or cross-contamination in sequencing [81,82]. We used appropriate methods (decontam-frequency) to identify and remove contaminants. However, future low biomass studies could improve upon our methods further by including negative controls, allowing for comparison of decontam methods across (frequency versus prevalence) sequencing types (short- versus long-read). The specific thresholds chosen to identify microbiome candidates in our study (relative abundance >0.1%, prevalence >10%) are not objectively established thresholds. However, they serve the purpose of focusing attention on a subset of the ASVs in our study that are more likely to be a legitimate part of the flea microbiome, and the thresholds are clearly described in the text so that our results can be properly interpreted.

We found clear evidence of primer bias in the short-read (V3/V4) data as indicated by detection of *Spiroplasma* in the long-read but not short-read data. While we did not find any clear evidence of similarly missed taxa by the long-read primer set, we cannot exclude this as a possibility. We also identified discrepancies between the taxonomic assignments made for *Bartonella* ASVs across the two databases used. This can likely be explained by differences in the databases, but full interrogation of the taxonomic assignment methodologies was beyond the scope of this manuscript. Future studies could benefit from further investigation of more specific genetic targets for *Bartonella* species identification. We did not identify whether fleas were fed or unfed in our study, but future studies might note this to help elucidate whether recent feeding plays a role in microbial composition.

By integrating short- and long-read sequencing, our study advances the understanding of the *C. felis* microbiome and its implications for flea-borne disease ecology. Our findings also demonstrate the importance of understanding that microbiome studies are limited by the bacteria (or other organisms) they select for, as was demonstrated by the lack of *Spiroplasma* detection in one sequencing modality and primer selection but not the other. Our findings support the inclusion of *Spiroplasma* as a core flea microbiome member, demonstrate the host-associated variation in flea microbiota, and reinforce the value of full-length 16S rRNA gene sequencing for species identification of potential pathogens. These insights are critical for improving surveillance and management strategies for flea-borne diseases in both veterinary and public health contexts.

## Supporting information

S1 Protocol Additional enrollment criteria for efficacy study. (DOCX)

S1 Table Read counts across samples at different stages in Methods. (XLSX)

S2 Table *Rickettsia* genome assemblies from NCBI Nov. 5, 2024. (TSV)

S3 Table *Bartonella* genome assemblies from NCBI Feb. 13, 2025. (CSV)

S4 Table Per-household overview of sample sources. (DOCX)

S5 Table Calculated phylogenetic distances for *Rickettsia.* (CSV)

S6 Table Calculated phylogenetic distances for *Bartonella.* (CSV)

S1 Fig *Rickettsia* phylogenetic tree. (SVG)

S2 Fig *Bartonella* phylogenetic tree. (SVG)

S1 File Full-length 16S sequences for ASVs 7 and 21. (FASTA)
