# Supplementary material for: Pathogenic bacterial species and the microbiome of cat fleas (Ctenocephalides felis) inhabiting flea-infested homes
Source: PLoS One. 2026 Jan 30;21(1):e0341824. doi: 10.1371/journal.pone.0341824 (PMC12857954; doi:10.1371/journal.pone.0341824)
Supplement: S1 Protocol — (DOCX) [file pone.0341824.s001.docx]

Inclusion criteria: **Evaluation of lotilaner to control flea populations on naturally infested cats in private residences in West Central FL, and to determine the prevalence of pathogens linked to those flea populations**

- Study design: observational
- Residential homes experiencing home flea infestations in the Tampa, Florida area were recruited to participate in a flea control study.
- Inclusion criteria
  - Home owners willing to participate in the study and adhere to study requirements
  - Owner (or qualified designate) signs the Owner Consent Form
  - Homes were categorized as “High” homes and “Low” homes according to the number of fleas present on cats and in environmental traps.
    - For Group 1 cats: “High” homes
      - at least 20 homes enrolled based on ≥5 fleas observed in area flea counts on at least one cat at the residence
      - At least five (5) fleas must be collected in a 16 – 24 hour period in two intermittent light flea traps
    - For Group 2 cats” “Low” homes
      - At least 10 homes enrolled based on <5 fleas observed in area flea counts on any cat at the residence, and <5 fleas collected in a 16 – 24 hour period in two intermittent light flea traps
      - Up to 10 homes with no fleas collected in a 16 – 24 hour period in two intermittent light flea traps and no overt evidence of flea infestations on any cat or in the home
  - At least one non-fractious cat and up to nine (9) additional cats or dogs at the residence (i.e., maximum total of 10 pets, whether cats or dogs). All enrolled pets must be visually examined by a veterinarian and considered to be clinically healthy. Cats with minor ailments judged not to interfere with the study may be included. For Group 1 there must be at least one qualifying cat in a home for a household to be enrolled and remain in the study
  - All household dogs and cats must spend ≥ 12 hrs/day in the indoor premises
  - Owners must agree not to use any topical or premise flea control products during the study
  - Owners must agree not to bring any other cats or dogs into the household for the duration of the study
  - No cat or dog in the household can be pregnant or nursing
  - Cats qualifying for the study must be ≥8 weeks of age and ≥2.0 lbs; dogs >8 weeks & > 4.4 lbs.
  - Owners must complete a questionnaire concerning pet habits, visiting pets, previous flea treatments and personal observations around their residence concerning wildlife and feral cats
  - No residual on-animal or oral flea product previously applied within at least one month beyond the product’s labeled duration of activity
  - No premise treatments that could impact flea populations applied within the 6 weeks prior to enrollment
  - While homes may have up to 10 cats and/or dogs residing at the home, no more than 5 total animals will be enrolled. For all qualifying households, each dog and cat must be treated with *Credelio* (lotilaner) or *Credelio* CAT (lotilaner), respectively, whether or not enrolled to be included for study assessments. However, all qualifying and non-qualifying cats and dogs in each home will be treated with *Credelio* per label, and treatments recorded.
- Sample collection
  - Flea Population Assessments
    - Flea counts on animals
      - Fleas were collected from cats and dogs using a flea combing technique described in a previous study. [[9]](https://www.zotero.org/google-docs/?dQYJfO)
      - The flea population on each enrolled cat (no more than 5 cats in any home) was assessed using a visual area count methodology. Each of the following six regions was examined using 10 strokes of a standard flea comb. Combing will commence on the (1) back of the head behind the ears and extend along the dorsal midline to the tail. Combing then proceeded to either the (2) left or (3) right side of the cat from the axillary region to the posterior aspect of the cat’s body, including hair on the posterior of each leg, then to the alternate side. The next region included the (4) ventral aspect of the cat from chest to inguinal region, followed by the (5) ventral neck region from chin to chest and finally (6) top of the head between the ears.
      - Total number of fleas removed were counted and for Group 1 immediately placed back on the cat, except for fleas saved for pathogen testing. All collected fleas from Group 2 animals on Day 0 were removed since product efficacy was not evaluated in this group. The subset of fleas taken on Day 0 was based on the level of infestation of each cat:
        - 5-9 fleas: No fleas removed
        - 10-14 fleas: 3 fleas removed
        - 15-24 fleas: 6 fleas removed; later split into two (2) groups of three (3) fleas
        - 25-49 fleas: 9 fleas removed; later split into three groups (3) of three (3) fleas
        - 50+ fleas: 12 fleas removed; later split into four groups (4) of three (3) fleas
      - All collected fleas were placed in a plastic bag with collected hair, and clearly identified with the household label.
      - The flea population on each dog was assessed using a visual area count methodology. Area counts were performed at five locations on each animal: dorsal midline, tail head, left lateral, right lateral, and inguinal region. Area counts were limited to one minute per location and conducted by parting the hair and examining the skin using both hands until the area was covered.
  - Environmental flea counts
    - The numbers of adult fleas emerging in each home were assessed using intermittent light traps described in a previous study. [[10]](https://www.zotero.org/google-docs/?SBhrPz)
    - One trap was placed in each of at least two rooms for approximately 16 to 24-h. Room selection was based on where the cat(s) spend most of their time or where owners had observed fleas.
    - Additional rooms had traps placed at the discretion of the Study Investigator.
    - All fleas were saved in 70% ethanol for later processing. Each day, fleas were counted and recorded, removed from the trap and placed into a tube of ethanol, labeled with house study number and trap location. Each vial was sealed with parafilm and placed into the corresponding slot of the provided box. Once rooms were selected, the traps were returned to those rooms in the same location at every counting period. Fleas collected on the adhesive pads of the traps were counted, characterized by microscopic observation as to species, and recorded on the Environmental Trap Counts form.
  - Schedule for collection of flea count samples
    - Group 1 area (on-animal) and environment flea counts were conducted on Days 0, 7, 14, 21, then once between Days 28 – 30, 40 – 44, 56 – 60 and 82 – 86.
    - Group 2 area and environment flea counts were conducted on Day 0 then once between days 28 – 30, 56 – 60 and 82 – 86.
  - Collection and storage of flea dirt
    - On Day 0 or -1, and then at the final visit (Day 82 – 86) each household was examined for flea feces and flea eggs (and larvae) where the animals were said to spend the majority of their time. Suitable collection areas were required to have signs of pet dander and or flea feces, but could be predominantly owner-reported as areas where the animal spends the majority of its time. Additional locations (e.g., bedrooms, TV area) for collection of flea debris were added at the discretion of the Investigator. A 1-foot square (1 ft2) area was scraped using a wooden tongue depressor while a vacuum fitted with a Zefon microvac cassette with a 0.45μm filter ran alongside to collect the dust. The filter was then removed and placed into a 15ml Falcon tube until ready to process for DNA extraction.
    - Fleas were pooled into groups of three based on home and animal or home and trap location, respectively. Fleas were snap frozen with liquid nitrogen and crushed using a sterile pestle. Whole genomic DNA was extracted using a commercial DNA extraction kit. Extracted DNA was stored at -20^o^C until testing.
    - Filters with flea dirt/dust on them had 1ml of genomic lysis buffer added to the tube and then were incubated at 56^o^C for 4 hours while rocking. After the 4 hours, the lysis buffer solution was poured into a clean tube and used for DNA extraction as described above. The remaining filter paper was saved at -20^o^C until completion of the project.
