# Supplementary material for: Pathogenic bacterial species and the microbiome of cat fleas (Ctenocephalides felis) inhabiting flea-infested homes
Source: PLoS One. 2026 Jan 30;21(1):e0341824. doi: 10.1371/journal.pone.0341824 (PMC12857954; doi:10.1371/journal.pone.0341824)
Supplement: S4 Table — (DOCX) [file pone.0341824.s005.docx]

**Overview of fleas sequenced evaluating the microbiome of *C. felis* fleas from 3 different sources**

| **House** | **Cat (Number of pools sequenced)** | **Dog  (Number of pools sequenced)** | **Traps (Number of pools sequenced)** |
| --- | --- | --- | --- |
| **1** | 1 (1)  2 (3) | 0 | 1 (1)  2 (4) |
| **4** | 1 (2) | 0 | 0 |
| **7** | 1 (2) | 0 | 0 |
| **9** | 1 (1) | 0 | 0 |
| **10** | 1 (3) | 0 | 0 |
| **11** | 1 (3) | 0 | 1 (4)  2 (3) |
| **13** | 1 (1) | 0 | 0 |
| **14** | 1 (2) | 1 (3)  2 (1) | 1 (2) |
| **15** | 1 (2)  2 (1) | 0 | 1 (4) |
| **17** | 1 (1) | 1 (4) | 1 (4) |
| **19** | 1 (1) | 0 | 1 (1)  2 (1) |
| **20** | 1 (1) | 0 | 1 (2)  2 (3) |
| **21** | 1 (1) | 0 | 0 |
| **23** | 1 (3) | 0 | 1 (4)  2 (4) |
| **24** | 1 (1) | 0 | 0 |
| **26** | 0 | 0 | 1 (1) |
| **28** | 1 (4)  2 (1)  3 (4) | 0 | 1 (4) |
| **29** | 1 (1) | 0 | 0 |
| **30** | 1 (2)  2 (1)  3 (2) | 1 (4) | 1 (3)  2 (4) |
| **32** | 1 (1) | 0 | 1 (4)  2 (3) |
| **35** | 1 (3)  2 (1) | 0 | 1 (2)  2 (2) |
| **37** | 0 | 1 (4) | 1 (3)  2 (3) |
| **41** | 1 (1)  2 (1) | 1 (3) | 1 (1)  2 (2) |
| **42** | 0 | 0 | 1 (1)  2 (4) |
| **43** | 1 (1) | 0 | 0 |
| **44** | 1 (1) | 0 | 1 (4)  2 (4) |
| **45** | 1 (2) | 0 | 0 |
| **48** | 1 (1) | 0 | 0 |
| **49** | 1 (1) | 0 | 0 |
| **50** | 1 (3)  2 (3) | 0 | 1 (4)  2 (4) |
| **51** | 1 (4) | 1 (3) | 1 (2) |
| **52** | 1 (3) | 0 | 1 (4)  2 (4) |
| **56** | 1 (1) | 1 (3) | 1 (2) |
| **58** | 0 | 0 | 1 (4) |
| **60** | 0 | 0 | 1 (4)  2 (2) |
| **62** | 1 (3) | 0 | 1 (1)  2 (2) |
| Total Homes: 36 | 40 cats (75 pools) | 8 dogs  (25 pools) | 40 traps (115 pools) |
